# High dietary B_12_ is associated with reduced gut microbial B_12_ biosynthesis capacity and lower fecal short-chain fatty acids in healthy United States adults

**DOI:** 10.1080/19490976.2026.2718567

**Published:** 2026-08-21

**Authors:** Margaret A. Hilliard, Andrew Oliver, Stephanie M. G. Wilson, Setareh Shahab-Ferdows, Daniela Hampel, Brian J. Bennett, Lindsay H. Allen, Danielle G. Lemay

**Affiliations:** a Department of Nutrition, University of California, Davis, CA, United States; b USDA-ARS Western Human Nutrition Research Center, Davis, CA, United States; c Genome Center, University of California, Davis, CA, United States

**Keywords:** Diet, gut microbiota, SCFA, vitamin B_12_, machine learning

## Abstract

Vitamin B_12_ is acquired through the consumption of animal-source foods and supplements. In animal models, interventions with B_12_ and/or methionine influence fecal short-chain fatty acid (SCFA) concentration. Yet the relevance of dietary B_12_ to microbially produced SCFAs in humans is unknown. This study determined associations between dietary B_12_ and the gut microbiome in a deeply phenotyped cohort of healthy U.S. adults. Habitual diet and fecal shotgun metagenomes were integrated alongside measurements of fecal SCFAs, plasma SCFAs, and plasma B_12_ (*n* = 277). Vitamin B_12_ intake ranged from 2.4 to 1062 µg/day, and nearly all participants were B_12_ replete. Stratification of participants into adequate (2.4–8.51 µg/day) and high B_12_ intake (>8.51 µg/day) groups revealed the association of high intake with a reduction in bacteria capable of anaerobic B_12_ biosynthesis. High B_12_ intake was also associated with lower fecal SCFA concentrations even after controlling for fiber and methionine intake. Differences in microbial taxa between dietary groups were limited. However, machine learning models demonstrated the ability to predict fecal propionate and butyrate from microbial pathways in the adequate or no supplement groups, but not in the high intake or supplement groups. Our results indicate that dietary B_12_ greater than 8.51 µg/day may be associated with reduced microbial synthesis of B_12_ and lower fecal SCFA production.

## Graphical abstract

**Figure:**
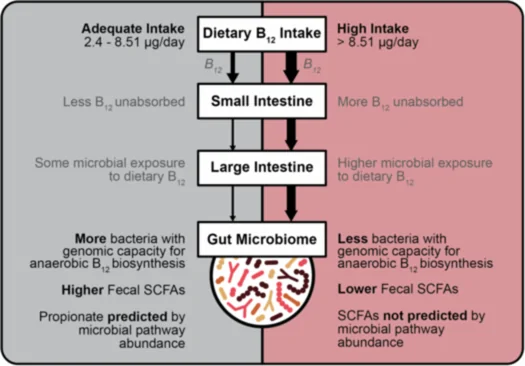

## Introduction

Animal source foods are the only natural source of vitamin B_12_ in the human diet. Fortified foods and supplements offer a practical way to increase B_12_ intake when intake of animal source foods is limited, whether due to food environment barriers, financial barriers, or dietary choices that restrict animal source foods.^1^ ^,^ ^2^ In the United States, where animal-source food intake is generally sufficient to meet the daily recommended intake of B_12_, many still fall short of meeting the estimated average requirements for several essential vitamins and minerals through diet alone.^3^ Consequently, nearly 58% of Americans take vitamin supplements, with usage highest among women and older adults.^4^

B_12_ is initially absorbed in the small intestine, and its bioavailability is approximately 50% from dietary sources, with decreases in absorption efficiency detected at higher pharmacologic doses.^5^ Dietary B_12_ evades host digestion through a multi-step process that culminates in receptor-mediated uptake in the ileum.^6^ Intrinsic factor-mediated host absorption is limited to a threshold of 1.5–2.5 µg per meal due to the delay in recycling of the endocytosed receptor back to the ileal enterocytes.^7^ Thus, with higher B_12_ intake, a greater fraction of dietary B_12_ bypasses small intestinal absorption and passes to the large intestine for modification and utilization by the gut microbiota.^8^ ^,^ ^9^ In the colon, B_12_ and related cobamide structures are ecologically important to the gut microbiota as co-factors to microbial enzymes involved in carbon, nitrogen, and nucleotide metabolism, as well as fermentation of diet- or mucus-derived monosaccharides.^9-12^ B_12_ also functions as a co-factor in microbial short-chain fatty acid (SCFA) production. Specifically, B_12_ is a co-factor in the propanediol and succinate pathways of propionate synthesis as well as acetate synthesis via the Wood–Ljungdahl pathway.^13-16^

Recent attention has been given to the effects of dietary methionine restriction on, for example, metabolic health^17^ ^,^ ^18^ and improved lifespan.^19^ Moreover, previous research in animal models has indicated that excess dietary methionine may inhibit microbial SCFA production,^20^ resulting in a decrease in these health-promoting metabolites.^21^ ^,^ ^22^ Yet the dietary sources and eating patterns that provide methionine are often rich in B_12_ as well. In vitro work investigating the relationship between B_12_ and SCFAs found mixed results that suggested an association dependent on gut microbial composition.^23^ In the present work, we analyzed the relationship between dietary B_12_ and fecal SCFAs in healthy humans, while controlling for dietary methionine and other person-specific factors that likely influence fecal SCFA abundance.

Despite the known functions of B_12_, it remains unclear if high dietary B_12_ intake impacts the taxonomic structure and functional capacity of the gut microbiome in humans. A systematic review linking dietary B_12_ to the gut microbiome highlighted the lack of human studies using metagenomic approaches.^24^ Our study addresses that gap by integrating dietary data with fecal shotgun metagenomes and fecal SCFA measurements from healthy U.S. adults in an observational cohort. Specifically, we tested the hypothesis that higher B_12_ intake is associated with a reduction of microbially produced fecal SCFAs and a reduction in anaerobic B_12_ biosynthesis capacity in the gut microbiome. After hypothesis testing, we used feature engineering and machine learning to examine associations between gut microbial taxa and metabolic pathways with fecal SCFA concentrations.

## Materials and methods

### Study design and participants

This work analyzes the USDA Nutritional Phenotyping Study, an observational, cross-sectional study that enrolled 393 healthy adult participants balanced by age (18–65 y), sex, and BMI (18.5–39.9 kg/m^2^) over the course of 4 y.^25^ Exclusion criteria included minor surgery, hospitalization, or antibiotic therapy in the previous 4 weeks, major surgery within the past 16 weeks, egg allergy, pregnancy or lactation, and diagnosed chronic disease treated with medication. The study protocol was approved by the Institutional Review Board at the University of California, Davis, and was prospectively registered on Clinicaltrials.gov (NCT02367287). All participants for whom the shotgun fecal metagenomes were sequenced and both plasma B_12_ and fecal and/or plasma SCFAs were quantified were included in this analysis. Plasma B_12_ was the most limiting analyte. In addition, we excluded participants who consumed less than the daily recommended intake of B_12_ (2.4 µg/day) based on responses from dietary assessments (*n* = 10). The stepwise reduction in participants from the parent study to the final analytic sample of *n* = 277 is shown in Supplemental Figure 1. The subset of participants in our analyses retained balanced recruitment of age and sex, while recruitment from the highest BMI category (≥30  kg/m^2^) was lower than that from the other two BMI categories (*p* = 0.01; Supplemental Table 1).

### Dietary assessment

Given that our outcomes of interest focused on microbial structure and function, we utilized habitual dietary estimates rather than recent intake, as habitual intake better reflects long-term B_12_ consumption and was therefore more likely to reveal stable microbial patterns associated with diet. Habitual intake of B_12_ from foods and supplements was estimated using the Block 2014 Food Frequency Questionnaire (FFQ; NutritionQuest, Berkeley, CA, USA). The FFQ estimates the habitual intake of 127 foods and beverages over the previous year. The questionnaire was filled out electronically under the supervision of trained personnel. Measuring cups and spoons were used as visual aids for determining portion sizes.

For our analyses, we stratified participants into four dietary groups: individuals whose B_12_ intake was in the upper 50th percentile (>8.51 µg per day, herein ‘high intake group’), lower 50th percentile (2.40–8.51 µg per day, herein ‘adequate intake group’), individuals who consumed non-zero B_12_ in the form of a supplement (herein ‘supplement use group’, *n* = 121), and those who did not consume B_12_ in the form of a supplement (‘no supplement use group’, *n* = 156). Other dietary variables from the FFQ included in our analyses were soluble dietary fiber and dietary methionine (both in grams/day). Baseline participant characteristics remained similar between the vitamin B_12_ intake groups and the supplement use groups (Supplemental Tables 2 and 3).

### Plasma sample collection

Fasting blood specimens were collected using Vacutainer® heparin tubes (BD, Franklin Lakes, NJ, USA). After 30 min on wet ice, plasma was obtained by centrifugation at 1300 × g at 4 °C for 10 min. The plasma was immediately aliquoted and stored in Cryo-Store® vials at −80 °C until analysis.

### Quantification of plasma B_12_ concentration

B_12_ concentrations in the plasma were analyzed using the cobas® e411 immunoassay analyzer employing the Elecsys® Vitamin B_12_ II kit (Roche Diagnostics, Indianapolis, IN, USA) according to the manufacturer’s protocol. To ensure precision and accuracy in each analytical batch, we included the Roche PreciControl Varia measured at levels 0, 1, and 2, as well as an internal UTAK control (UTAK Laboratories Inc., Valencia, CA, USA).

### Quantification of plasma SCFAs

Plasma SCFAs were measured in EDTA-treated plasma samples at the Columbia University Medical Center Biomarkers Core Laboratory using ultra-performance liquid chromatography‒tandem mass spectrometry as previously described.^26^

### Stool collection

Sanitary collection supplies were provided, and participants received an email reminder 3–4 d prior to the scheduled site visit. Each participant was asked to provide a single stool sample and transport the sample on cold packs to the research center as soon as possible for same-day processing. The specimen was homogenized using a Stomacher® paddle blender, frozen, and stored in Cryo-Store® vials at −80 °C until analysis.

### Quantification of fecal SCFAs

Fecal SCFAs were measured via gas chromatography‒mass spectrometry as previously described from approximately 150 mg of wet stool.^26^

### Fecal DNA extraction and metagenomic sequencing

DNA from fecal samples was extracted using the ZymoBiomics DNA Miniprep kit (Zymo Research, Irvine, CA, USA), as previously described.^27^ Library preparation and sequencing were performed by the DNA Technologies & Expression Analysis Core Laboratory at the University of California, Davis Genome Center. Library insert sizes were 250–280 bp, and the exact size selection of DNA fragments was performed with a PippinHT instrument (Sage Science, Beverly, MA, USA). Libraries were combined into a single pool for each sequencing batch and sequenced in a 2 × 150-bp format on the NovaSeq6000 platform (Illumina, San Diego, CA, USA), with an S4 flow cell resulting in an approximately 20-bp overlap in the reads. Initially, 290 samples were sequenced and analyzed.^27^ Later an additional 40 samples were sequened and analyzed using identical methods.^26^ A combination of these previously described fecal shotgun metagenomes were analyzed in this publication (**Supplemental Figure 1**).

### Metagenomic sequencing pre-processing

Metagenomic reads were processed as previously described.^27^ Briefly, BMTagger was used to remove reads aligning to the human genome version GRCh38.p13 from all samples.^28^ ^,^ ^29^ Afterward, Trimmomatic version 0.33 was used to remove TruSeq paired-end adapters and reads were trimmed with a sliding window of 4 bp, a minimum average quality of 15, and a minimum length of 99 bp.^30^ Duplicated reads were then removed using FastUniq version 1.1 with default settings.^31^ The resulting paired-end reads were assembled using FLASH version 1.2.11 with the overlapping length set between 10 bp to 100 bp and the mismatch ratio set at 0.1.^32^ Code to reproduce the pre-processing of metagenomic sequences from this work can be found at https://github.com/dglemay/ARG_metagenome.

### Read-based taxonomic and functional profiling of the microbiome

Merged reads output from FLASH were used as input to MetaPhlAn v4.0.6 against the vOct22 database, using default parameters and the --add_viruses flag.^33^ Prior to running the differential abundance analysis and machine learning (ML) models using these taxonomic data, the default MetaPhlAn taxonomy table was converted to Genome Taxonomy Database (GTDB) format using the utility script (sgb_to_gtdb_profile.py) provided by the MetaPhlAn developers.

Merged reads output from FLASH were also used as input to HUMAnN version 3.7 using the default parameters and the flag --search-mode uniref90.^34^ The ChocoPhlAn database used was v201901_v31, and the uniref90 database was v201901b_full. Pathway abundance files were merged and normalized to copies per million using the humann_renorm_table command.

### Differential abundance analysis

The relative abundances of microbial taxa were used to construct multivariable associations with linear models using the MaAsLin3 package in R.^35^ We aimed to identify taxa that were differentially abundant between our dietary groups based on total B_12_ intake and the use of B_12_ supplements. To account for differences in the host-indigestible nutrients available for utilization by the gut microbiome, we added dietary soluble fiber intake, as estimated by the FFQ, to all model formulas. In addition, the arguments min_prevalence and min_abundance were set to 0.25 and 0.001, respectively. All other arguments were set to default.

### Assembly-based functional profiling of the microbiome

Reads were individually assembled using MEGAHIT v1.2.9.^36^ Bowtie2 v2.5.2 was used to recruit metagenomic reads to contigs, and samtools v1.19.2 was used to convert SAM files to BAM files.^37^ ^,^ ^38^ MetaBAT 2 v2.18 was used to generate read depth files from the sorted BAM files, and to subsequently bin contigs.^39^ Bin reconstruction quality was evaluated using CheckM2 v1.1.0.^40^ For our assembly-based analyses, we used medium-quality draft metagenome-assembled genomes (MAGs), defined as bins that were estimated as being greater than or equal to 50% complete and less than 10% contaminated.^41^

Metabolic pathway reconstruction and functional profiling were performed using anvi’o v8.^42^ MAG fasta files were imported and reformatted for use with anvi’o, then open reading frames were predicted using anvi-gen-contigs-database. Functional annotations were performed with anvi-run-kegg-kofams to assign genes to metabolic pathways using the v2023-09-22 KEGG snapshot. Annotated MAGs were then input to anvi-estimate-metabolism to predict the presence of metabolic pathways and summarize potential metabolic functions using default parameters.^43^

The proportion of microbial taxa with the genomic capacity for anaerobic B_12_ biosynthesis was quantified using the presence of two composite KEGG modules, M00924 and M00122. A list of queried enzymes can be found in Supplemental Table 4, which were used to create a custom metabolic module for use with anvi’o.^44^ ^,^ ^45^ A pathway was considered complete if ≥90% of the pathway was present in a MAG, given the following pathway definition: (K02302,(K02303,K13542,K00589,K02496,K13543) (K02304,K24866)) (K02190,K03795,K22011) (K03394,K13540) (K05934,K13541,K21479) K05936 (K02189,K13541) K02188 K05895 (K02191,K03399,K00595) K06042 K02224 (K00798,K19221) K02232 (K02225,K02227) K02231 K00768 (K02226,K22316) K02233. To examine the associations between dietary B_12_ intake and microbial B_12_ biosynthetic potential, the results were aggregated by subject and were visualized to represent the proportion of taxa with a complete pathway relative to the total number of recovered MAGs (per subject) between our dietary groups based on total B_12_ intake and the use of B_12_ supplements.

### Machine learning

Random forest regression models were used to assess the predictive capacity of gut microbial composition and pathway abundances on the concentration of fecal acetate, propionate, and butyrate. Each of our models included the covariate features of age, sex, and BMI, alongside a summary dietary feature from the FFQ that estimated soluble dietary fiber (in grams) consumed per day. To explore how B_12_ intake may shape microbial metabolism, we stratified participants into four dietary groups: individuals whose B_12_ intake was in the upper 50th percentile (>8.51  µg per day, herein referred to as the ‘high intake group’), lower 50th percentile (<8.51 µg per day, herein referred to as the ‘adequate intake group’), individuals who consumed non-zero B_12_ in the form of a supplement (herein referred to as the ‘supplement use group’, *n* = 101), and those who did not consume B_12_ in the form of a supplement (‘no supplement use group’, *n* = 146). From each of these dietary groups, predictive ML models of each fecal SCFA were built using either the merged MetaPhlAn table or the merged and normalized HUMAnN table. Across the four dietary groups, this approach yielded 24 models (Supplemental Table 5).

The implementation of random forest regression models was done using DietML for microbiome pathway abundance models or TaxaHFE-ML for microbiome composition models (https://github.com/aoliver44/taxaHFE).^46^ DietML and TaxaHFE-ML have many optional arguments; as such, tuning was performed by using a random search grid (*n* = 500 combinations) to maximize R^2^ (of the DietML or TaxaHFE-ML model), defining the following parameters: corr_level, n_perm, train_split, folds, metric, tune_length, tune_time, tune_stop, prevalence, abundance, and superfilter. Metabolite-specific tuning was done using full data sets, regardless of dietary intake. Once we identified the program arguments that yielded the top-performing models, we split the data into the four categories of B_12_ intake and re-ran those top models 100 times using randomly generated seeds to account for variability in initial test-train splits. The test data scores were averaged. We calculated feature importance for the highest-performing models based on R^2^; additionally, these models had lower MAE compared to the null model (the null model is one that predicts the response using just the mean of the response calculated with the training data). Shapley Additive exPlanations (SHAP) values were calculated and used to explain feature importance.

### Statistical analysis

All the statistical analyses were conducted in R version 4.4.3 with a significance threshold of *α* = 0.05.^47^ Visualizations were done using the ggplot2 and patchwork packages.^48^ ^,^ ^49^ Chi-square goodness-of-fit tests were used to evaluate whether the categories of age, sex, and BMI were evenly distributed in study participants. Comparisons of age and BMI between categories of dietary intake and supplement use were done using independent t-tests, and chi-square goodness-of-fit tests were used for sex comparisons. Tests were implemented using the gtsummary package in R.^50^

Plasma B_12_ concentration, dietary B_12_, and dietary methionine were assessed for residual normality and transformed accordingly using the bestNormalize package.^51^ Group differences in plasma B_12_ concentrations, dietary B_12_, and dietary methionine between the categories of dietary intake and supplement use were evaluated with Mann–Whitney U tests using the rstatix package.^52^ Associations between plasma B_12_ and predictors (age, sex, BMI, dietary B_12_) were assessed using multivariable linear regression using the car package in R.^53^ Similarly, associations between dietary methionine and predictors (age, sex, BMI, soluble fiber, and dietary B_12_) were assessed using multivariable linear regression using the car package.

Microbial alpha diversity was computed using the vegan package in R and modeled against the categories of dietary intake and supplement use as predictors using linear regression.^54^ Beta diversity analyses were done using the adonis2 function in the vegan package using 999 permutations.

Partial Spearman correlations were computed using the ppcor package in R, which rank-transformed variables (dietary B_12_, methionine, fecal SCFAs, age, sex, BMI, and soluble fiber intake) prior to estimating partial correlations.^55^ Associations between dietary B_12_ and fecal SCFAs were modeled separately using Mann–Whitney U tests in the rstatix package and generalized linear models (Gamma distribution, log-link), which adjusted for age, sex, BMI, soluble fiber intake, and dietary methionine. Model fit was assessed with the DHARMa package,^56^ and estimated marginal means were back-transformed to the original scale using the emmeans package.^57^ Pairwise contrasts were computed using the contrast() function within the emmeans package, and associated *p*-values were FDR-adjusted.

Marginal slopes of dietary B_12_ were estimated using the emtrends() function from the emmeans package, providing covariate-adjusted estimates of the linear trend on the log scale. Two-sided *p*-values for slopes were computed using Wald z-tests. For visualization, model-adjusted fecal SCFA concentrations were generated across the observed range of normalized B_12_ intake using estimated marginal means on the response scale. The predicted lines of best fit and 95% confidence intervals were plotted alongside raw data to illustrate covariate-adjusted relationships between B_12_ intake and fecal SCFA concentrations.

As a preliminary test of group differences, Mann–Whitney U tests were conducted for each plasma SCFA against the categories of dietary intake and supplement use. Partial Spearman correlations were computed using the ppcor package in R, which rank-transformed variables (dietary B_12_, plasma SCFAs, age, sex, BMI, dietary methionine, and soluble fiber intake) prior to estimating partial correlations.^55^ Inspection of the lower tail of plasma butyrate and propionate distributions revealed evidence of left-censoring. Accordingly, Tobit regressions were implemented using the VGAM package in R for plasma propionate and butyrate with the lower censoring threshold set to the observed minimum for each analyte while adjusting for age, sex, BMI, dietary fiber, and methionine in the models.^58^ Linear regressions adjusting for age, sex, BMI, dietary fiber, and methionine that used a normalized response variable generated using the bestNormalize package were implemented to model associations between plasma acetate and the categories of dietary intake and supplement use. The fit of all regression models was assessed with the DHARMa package.^56^

Machine learning performance was evaluated using mean absolute error (MAE) and R^2^. For each model, the percent change in performance relative to a null model was calculated. Model performance was summarized as the mean ± standard error, calculated using the plotrix package.^59^ Model performance comparisons were conducted using a global Kruskal–Wallis test and Dunn’s post-hoc test using the FSA package.^60^ A Benjamini–Hochberg correction for multiple testing was applied to *p*-values, and statistical differences between ML models were visualized using compact letter displays with the multcompView package.^61^ Visualizations and statistical analyses reported in this paper are provided in a public GitHub repository at https://github.com/margarethilliard/VB12-analysis.

## Results

### Higher dietary B_12_ is associated with supplemented B_12_

**Figure 1.**
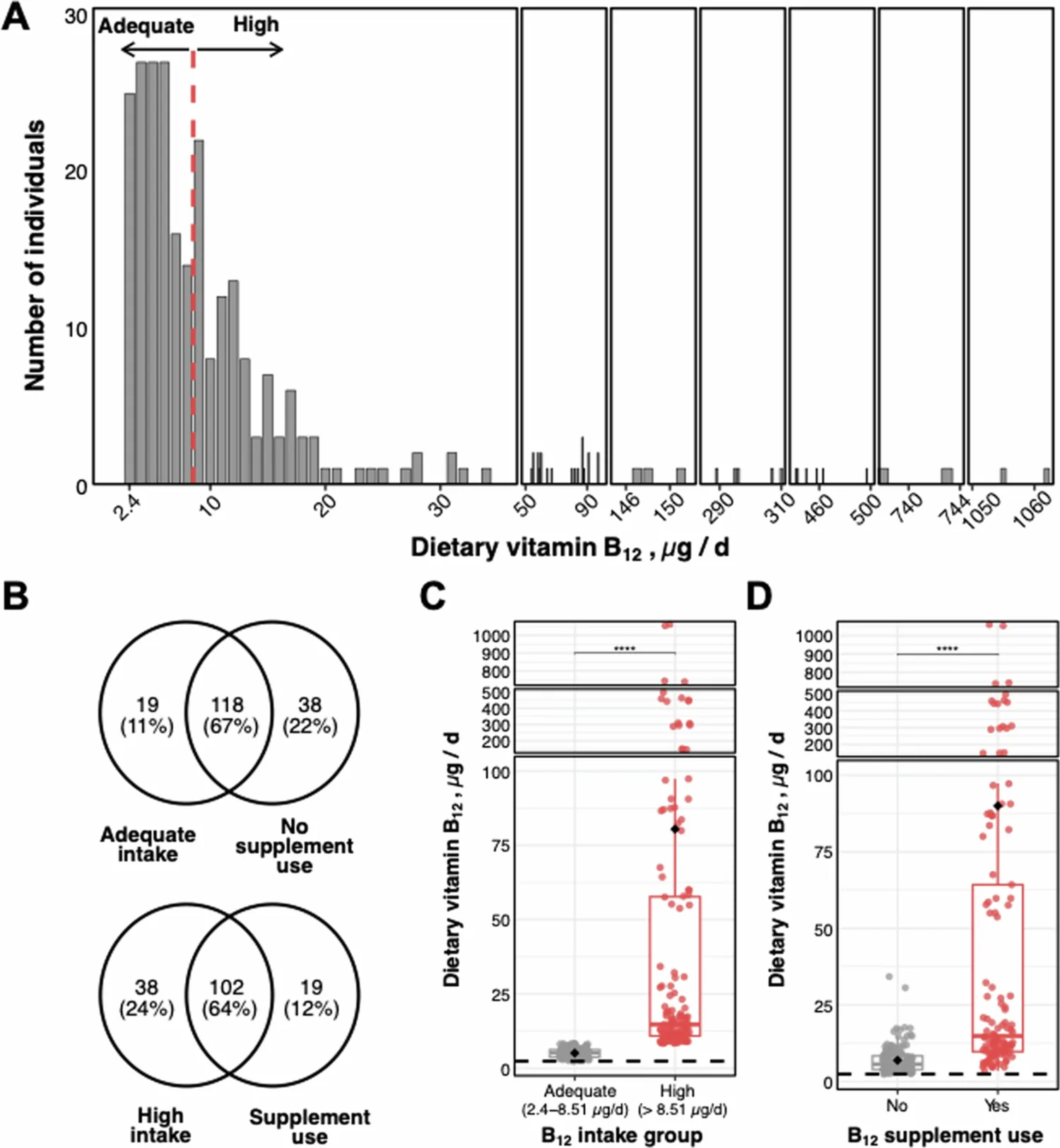
An overview of vitamin B_12_ intake in a healthy U.S. adult cohort. (A) The discrete distribution plot shows dietary B_12_, rounded to the nearest whole number, across 277 individuals. The vertical red dashed line indicates the median split (8.51 µg/day), which we used to define our adequate (2.4–8.51 µg/day) and high (>8.51 µg/day) intake groups. (B) Venn diagrams show the number of participants and the percentage overlap between dietary grouping variables based on total B_12_ intake and B_12_ supplement use. Boxplots comparing vitamin B_12_ intake based on (C) participant intake of vitamin B_12_: adequate intake: 2.40–8.51 µg/day, high intake: > 8.51 µg/day) or (D) use of vitamin B_12_ supplements (‘B_12_ supplement use’, *n* = 121; ‘no B_12_ supplement use’, *n* = 156). The upper and lower lines of the boxplots represent the upper and lower quartiles, while the middle line represents the median. The black dashed lines represent the recommended daily intake of vitamin B_12_ (2.4 µg/day), and the black diamonds represent the mean intake in each group. Group differences were determined using a Wilcoxon rank sum test, and significance is denoted by asterisks (*p < 0.05, **p < 0.01, ***p < 0.001; ns = not significant).

We estimated dietary intake of B_12_ (µg/day) based on foods and supplements recorded in the food frequency questionnaire from 277 healthy individuals (Supplemental Table 1 and Figure 1). Dietary B_12_ intake was positively skewed (Figure 1A) for our subset of participants who habitually consumed greater than the daily recommended intake of B_12_ (2.4 µg/day). We split participants into two groups based on their intake of B_12_ from foods and supplements: individuals whose B_12_ intake was in the upper 50th percentile (herein the ‘high intake group’) versus those in the lower 50th percentile (herein the ‘adequate intake group’). We also split participants into two groups based on their consumption of vitamin B_12_ supplements. We assessed B_12_ supplement use using the food frequency questionnaire and found that nearly 44% of participants consumed non-zero B_12_ in the form of a vitamin supplement (herein ‘supplement use group’, *n* = 121; ‘no supplement use group’, *n* = 156). We found that dietary B_12_ was higher on average in the high intake group compared to the adequate intake group (*p* < 0.001, Figure 1C), and in the supplement use group compared to the no supplement use group (*p* < 0.001, Figure 1D). The number of individuals who did not supplement B_12_ overlapped with the adequate intake group (*n* = 118, 67%; Figure 1B). Additionally, the number of individuals who use B_12_ supplements overlapped with high habitual B_12_ consumers (*n* = 102, 64%; Figure 1B). Thus, a majority of the high intake group used B_12_ supplements, and a majority of the adequate intake group did not use B_12_ supplements.

### Plasma B_12_ concentrations are associated with B_12_ intake from foods and supplements

**Figure 2.**
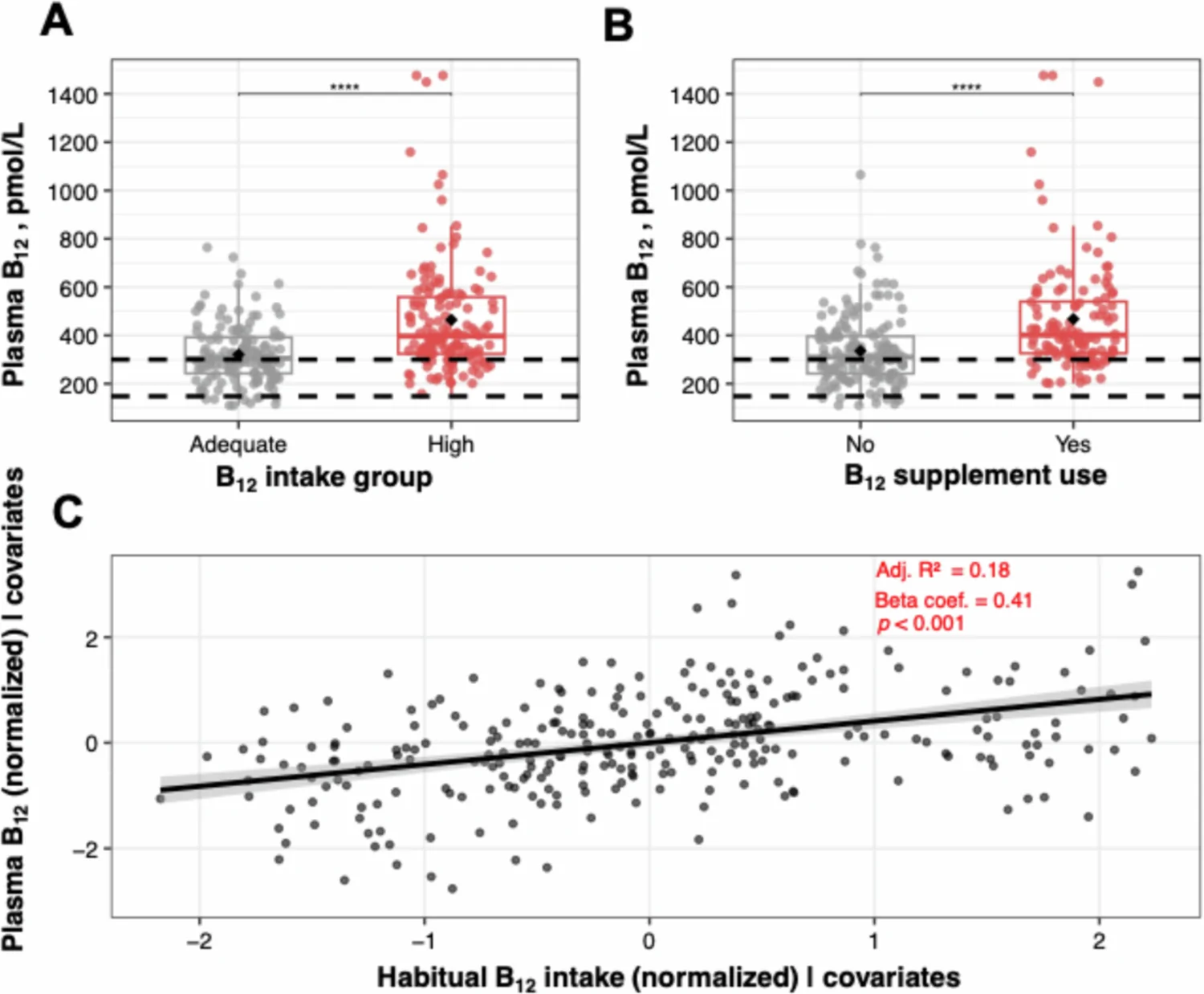
An overview of plasma B_12_ status in a healthy U.S. adult cohort. Boxplots comparing plasma vitamin B_12_ concentrations based on (A) participant intake of vitamin B_12_ (adequate intake: 2.40–8.51 µg/day, high intake: > 8.51 µg/day) or (B) use of vitamin B_12_ supplements (‘B_12_ supplement use’, *n* = 121; ‘no B_12_ supplement use’, *n* = 156). The upper and lower lines of the boxplots represent the upper and lower quartiles, while the middle line represents the median. The black dashed lines represent the replete range for plasma B_12_ (148–300 pmol/L), and the black diamonds represent the mean concentration in each group. Group differences were determined using a Wilcoxon rank sum test, and significance is denoted by asterisks (*p < 0.05, **p < 0.01, ***p < 0.001; ns = not significant). (C) Partial regression between the plasma B_12_ concentration and B_12_ intake, adjusting for age, sex, and BMI in the model. The black line represents the linear line of best fit, and the gray shading represents the 95% confidence intervals.

Plasma B_12_ concentrations were positively skewed and showed nearly all participants (>96%) were replete for plasma B_12_ (148–300 pmol/L; Figure 2A, B). We found that plasma B_12_ concentrations were higher on average in the high intake group compared to the adequate intake group (*p* < 0.001, Figure 2A), and in the supplement use group compared to the no supplement use group (*p* < 0.001; Figure 2B).

As expected, a partial regression adjusted for age, sex, and BMI revealed a strong positive relationship between plasma B_12_ concentration and B_12_ intake from diet and supplements (adjusted R^2^ = 0.18, *β* = 0.41, *p* < 0.001; Figure 2C). Age had a weak negative relationship with the plasma B_12_ concentration (partial regression *β* = −0.01, *p* = 0.01), while sex and BMI had no relationship (Supplemental Figure 2).

### B_12_ intake is weakly associated with changes in the proportion of bacterial taxa with genomic capacity for anaerobic B_12_ biosynthesis

**Figure 3.**
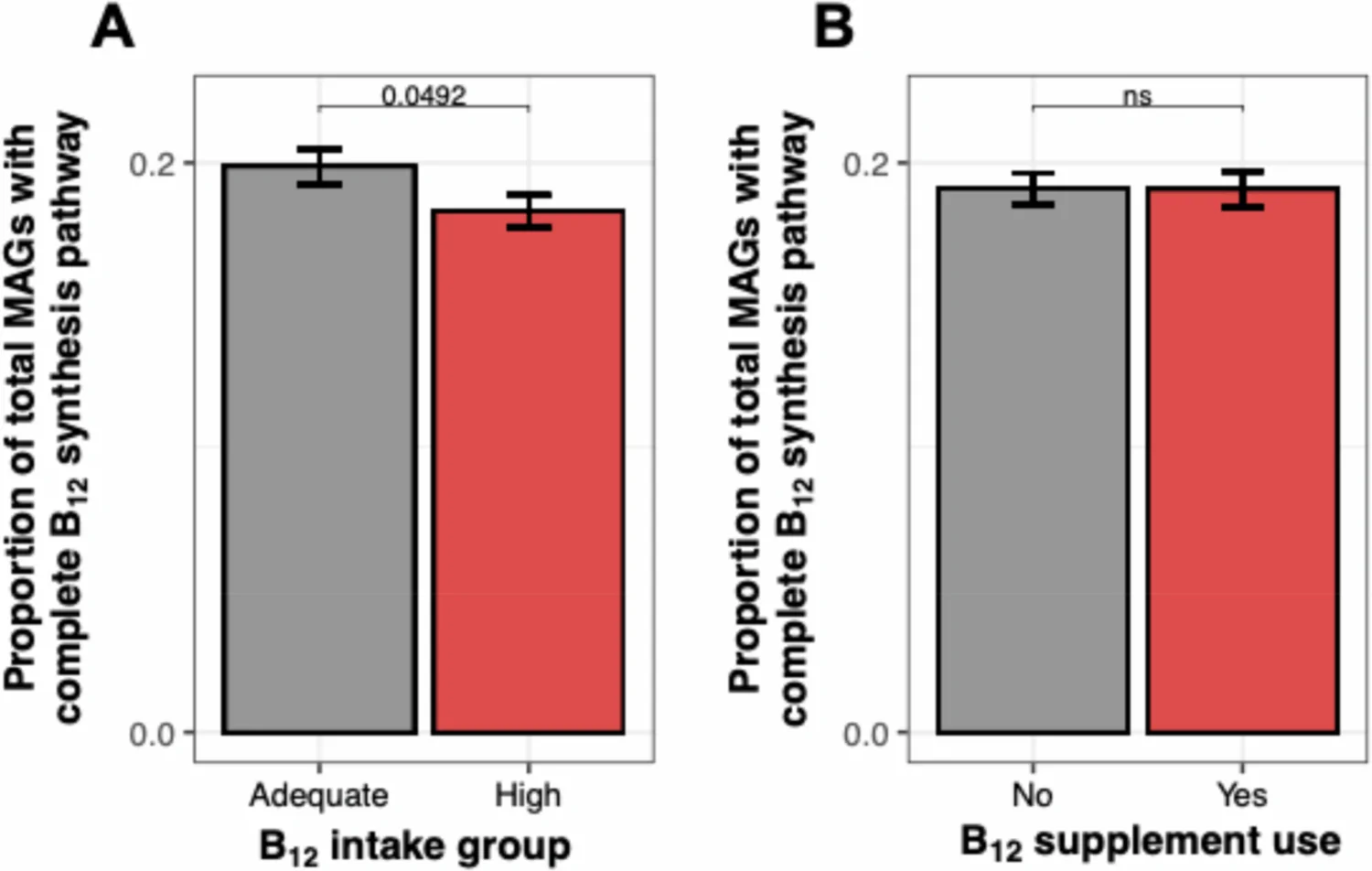
Differences in the proportion of microbial taxa with the genomic capacity for anaerobic vitamin B_12_ biosynthesis between vitamin B_12_ dietary groups. Anaerobic vitamin B_12_ biosynthesis was quantified from medium-quality draft metagenome-assembled genomes (MAGs) using the presence of two composite KEGG modules, M00924 and M00122. A pathway was considered complete ≥ 90% of the total KEGG modules were present in a MAG. The proportion of complete B_12_ biosynthesis pathways was calculated per subject based on the total number of MAGs. The bars represent the mean proportion of complete pathways across individuals in each group, and the error bars represent the standard error of the mean. (A) Proportion of complete B_12_ pathways based on participant B_12_ intake group (adequate intake: 2.40–8.51 µg/day, high intake: >8.51 µg/day). (B) Proportion of complete B_12_ pathways based on participant use of vitamin B_12_ supplements. Group differences were determined using a Wilcoxon rank sum test, and significance is denoted (ns = not significant).

We next tested the hypothesis that greater B_12_ intake would be associated with a community-wide reduction in the capacity for anaerobic B_12_ biosynthesis. Specifically, we asked if the proportion of microbial taxa encoding the complete anaerobic B_12_ biosynthesis pathway was different based on the habitual dietary habits of the host. Available read-based functional profiling tools (HUMAnN) quantify B_12_ salvage pathways, but lack comprehensive annotation for the anaerobic B_12_ biosynthesis pathway. Thus, we employed an assembly-based approach to detect the genomic capacity for anaerobic B_12_ biosynthesis that enabled the species-level detection of our function of interest. We found a reduction in the proportion of taxa with the genomic capacity for B_12_ biosynthesis among the high intake group (Figure 3A; *p* = 0.049). We found no difference in the mean proportion of total MAGs with complete B_12_ biosynthesis pathways based on B_12_ supplement use (Figure 3B).

### Greater B_12_ intake from food and supplements is associated with lower fecal SCFA concentrations

In animal models, diets high in the amino acid methionine have been implicated as being inhibitory to microbial fecal SCFA production. As animal source foods are high in both methionine and B_12_, which one is responsible for reduced SCFA production, and whether this relationship exists in humans, is unknown. In the current cohort, we found that dietary methionine and B_12_ were correlated with each other (partial Spearman’s rho = 0.22, *p* < 0.001). Interestingly, the relationship between dietary B_12_ and methionine was strengthened when analyzing only participants in the adequate intake group and no supplement use group, but disappeared when looking at the high intake group and the supplement use group (Supplemental Figure 3A, 3C). Similarly, we found that dietary methionine intake was higher on average in the high intake group (*p* < 0.001), but was not different based on B_12_ supplement use (*p* = 0.7, Supplemental Figure 3B, 3D). Thus, the high intake group and supplement users offer the ability to disentangle B_12_ and methionine with respect to associations with fecal SCFAs because these participants consumed high amounts of B_12_ without sustaining the positive relationship with dietary methionine intake that would otherwise be expected when consuming animal source foods. To our surprise, we found that dietary methionine was not correlated with fecal acetate (partial Spearman’s rho = 0.06, *p* = 0.4), propionate (partial Spearman’s rho = 0.08, *p* = 0.2), or butyrate (partial Spearman’s rho = 0.04, *p* = 0.5) after controlling for age, sex, BMI, soluble dietary fiber, and dietary B_12_. In contrast, we found that dietary B_12_ was negatively correlated with fecal acetate (partial Spearman’s rho = −0.2, *p* = 0.002) and propionate (partial Spearman’s rho = −0.15, *p* = 0.02) after controlling for age, sex, BMI, soluble dietary fiber, and dietary methionine. We did not find that dietary B_12_ was correlated with fecal butyrate (partial Spearman’s rho = −0.1, *p* = 0.08).

**Figure 4.**
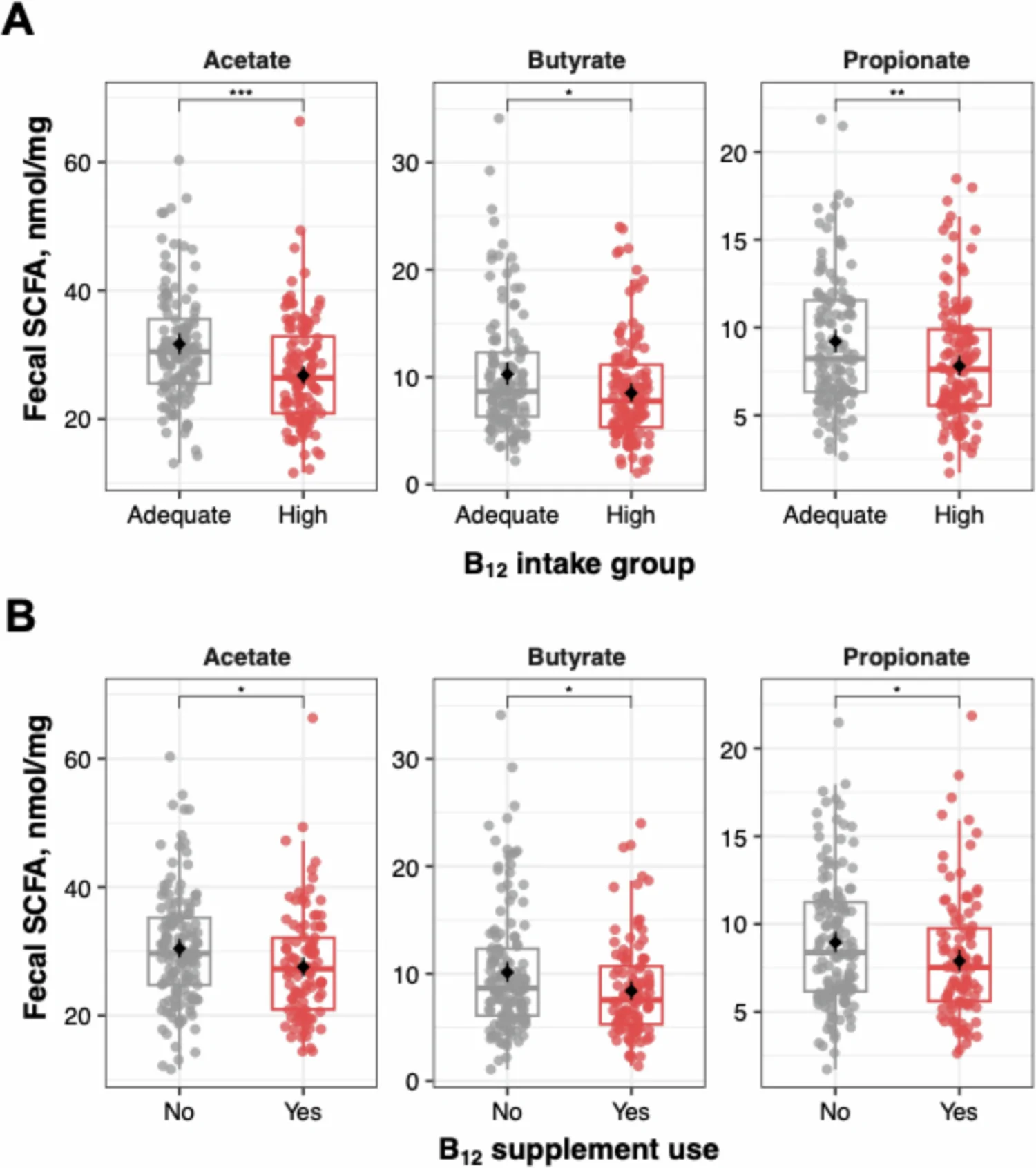
Fecal SCFA concentrations and their model-adjusted means between vitamin B_12_ dietary groups. Raw concentrations of fecal acetate, butyrate, and propionate are shown alongside the estimated marginal means (EMMs) predicted from generalized linear models adjusted for age, sex, BMI, dietary soluble fiber, and dietary methionine. (A) Fecal SCFA concentrations based on participant dietary intake of vitamin B_12_ (adequate intake group: 2.40–8.51 µg/day, high intake: >8.51 µg/day). (B) Fecal SCFA concentrations based on participant use of vitamin B_12_ supplements. The upper and lower lines of the boxplots represent the upper and lower quartiles, while the middle line represents the median. The black diamonds and vertical black lines represent the EMMs and the 95% confidence intervals, respectively. Significance of pairwise contrasts is denoted by asterisks (*p < 0.05, **p < 0.01, ***p < 0.001; ns = not significant) after false discovery rate correction.

We found that fecal SCFAs were significantly different between B_12_ intake groups and based on supplement use (Supplemental Table 6). Thus, we extended the analyses of B_12_ and fecal SCFAs to the categories of dietary intake and supplement use by using generalized linear models that included the covariates age, sex, BMI, soluble fiber intake, and dietary methionine. Pairwise contrasts of estimated marginal means (EMMs) revealed that model-adjusted SCFA concentrations were lower in the high B_12_ intake group (Figure 4A) and in the supplement use group (Figure 4B). The participants in the adequate intake group had significantly higher adjusted mean concentrations of fecal acetate (EMM = 31.7 nmol/mg, 95% CI [30.1, 33.3]), propionate (EMM = 9.2 nmol/mg, 95% CI [8.6, 9.9]), and butyrate (EMM = 10.3 nmol/mg, 95% CI [9.3, 11.4]) compared to the high intake group (acetate: EMM = 26.8 nmol/mg, 95% CI [25.5, 28.2]; propionate: EMM = 7.8 nmol/mg, 95% CI [7.3, 8.4]; butyrate: EMM = 8.5 nmol/mg, 95% CI [7.7, 9.4]). All three pairwise contrasts were statistically significant (acetate: ratio = 1.2, 95% CI [1.1, 1.3], FDR-adj. *p* < 0.001; propionate: ratio = 1.2, 95% CI [1.1, 1.3], FDR-adj. *p* = 0.007; butyrate: ratio = 1.2, 95% CI [1.03, 1.4], FDR-adj. *p* = 0.02). A consistent pattern was observed for B_12_ supplement use. Participants that did not use supplements had higher adjusted mean concentrations of acetate (EMM = 30.4 nmol/mg, 95% CI [29.0, 31.9]), propionate (EMM = 9.0 nmol/mg, 95% CI [8.4, 9.6]), and butyrate (EMM = 10.1 nmol/mg, 95% CI [9.2, 11.1]) compared to supplement users (acetate: EMM = 27.6 nmol/mg, 95% CI [26.1, 29.1]; propionate: EMM = 7.9 nmol/mg, 95% CI [7.3, 8.5]; butyrate: EMM = 8.4 nmol/mg, 95% CI [7.5, 9.3]), with all contrasts reaching statistical significance (acetate: ratio = 1.1, 95% CI [1.03, 1.2], FDR-adj. *p* = 0.01; propionate: ratio = 1.1, 95% CI [1.02, 1.3], FDR-adj. *p* = 0.02; butyrate: ratio = 1.2, 95% CI [1.04, 1.4], FDR-adj. *p* = 0.02).

**Figure 5.**
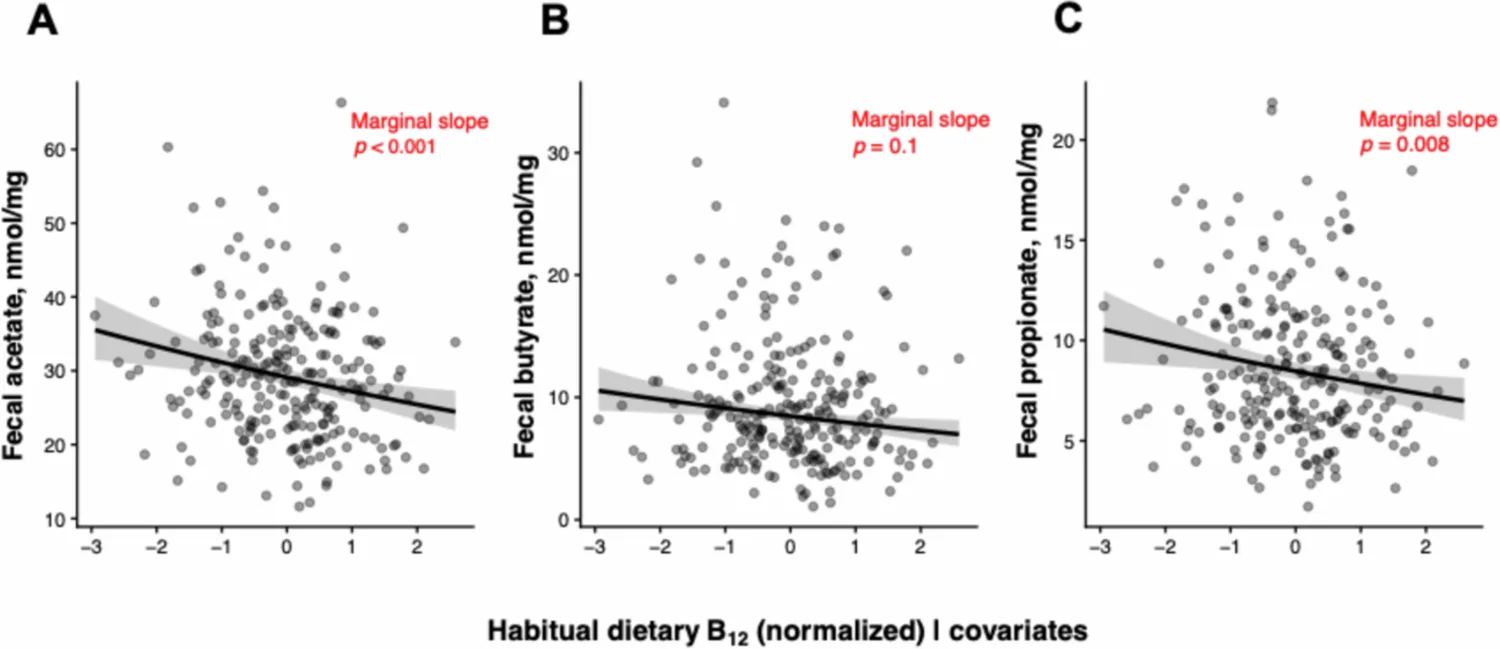
Estimated marginal slopes of predicted fecal SCFA concentrations across the observed range of normalized B_12_ intake. Model-adjusted estimates of fecal (A) acetate, (B) butyrate, and (C) propionate concentrations predicted from generalized linear models adjusted for age, sex, BMI, dietary soluble fiber, and dietary methionine. The lines represent adjusted mean values, the shaded areas indicate the 95% confidence intervals, and the displayed *p*-values indicate the significance of a two-tailed Wald z-test.

Considering these findings, we extended our hypothesis-driven inquiry to test if there was a negative dose response from B_12_ from foods and supplements on fecal SCFA concentrations. Additional generalized linear models were constructed that adjusted for age, sex, BMI, soluble fiber intake, and dietary methionine. Estimated marginal slopes of fecal SCFA concentrations across normalized observations of dietary B_12_ levels were calculated. Consistent with our previous results, the average marginal effect of dietary B_12_ on fecal acetate was negative (*β* = −0.07, SE = 0.02, CI [−0.1, −0.03], *p* < 0.001; Figure 5A). Similarly, the average marginal effect of dietary B_12_ on fecal propionate was negative (*β* = −0.07, SE = 0.03, CI [−0.1, −0.02], *p* = 0.008; Figure 5C). However, the average marginal effect of dietary B_12_ on fecal butyrate was not significantly different than zero (*β* = −0.06, SE = 0.04, CI [−0.1, 0.02], *p* = 0.1; Figure 5B).

### Dietary B_12_ is not associated with plasma SCFA concentrations, microbial diversity, or differences in compositional abundance or prevalence

Given that microbially-derived SCFAs can enter systemic circulation, we investigated whether the differences we observed in fecal SCFAs based on B_12_ intake from food and supplements would extend to associations with plasma SCFA concentrations. However, plasma acetate, propionate, and butyrate concentrations were not associated with B_12_ intake, dietary intake group, or supplement use (Supplemental Table 7).

There were also no associations between dietary B_12_ intake, or the four categories of B_12_ intake, with alpha or beta diversity of the microbiome (Supplemental Table 8). Despite this, we conducted a differential abundance analysis to examine differences in taxonomic abundance and prevalence based on B_12_ intake. We found no differences in compositional abundance or prevalence based on our dietary intake groups or by supplement use.

### Fecal propionate concentrations were predicted by microbial and pathway abundance in participants with lower B_12_ intake

**Figure 6.**
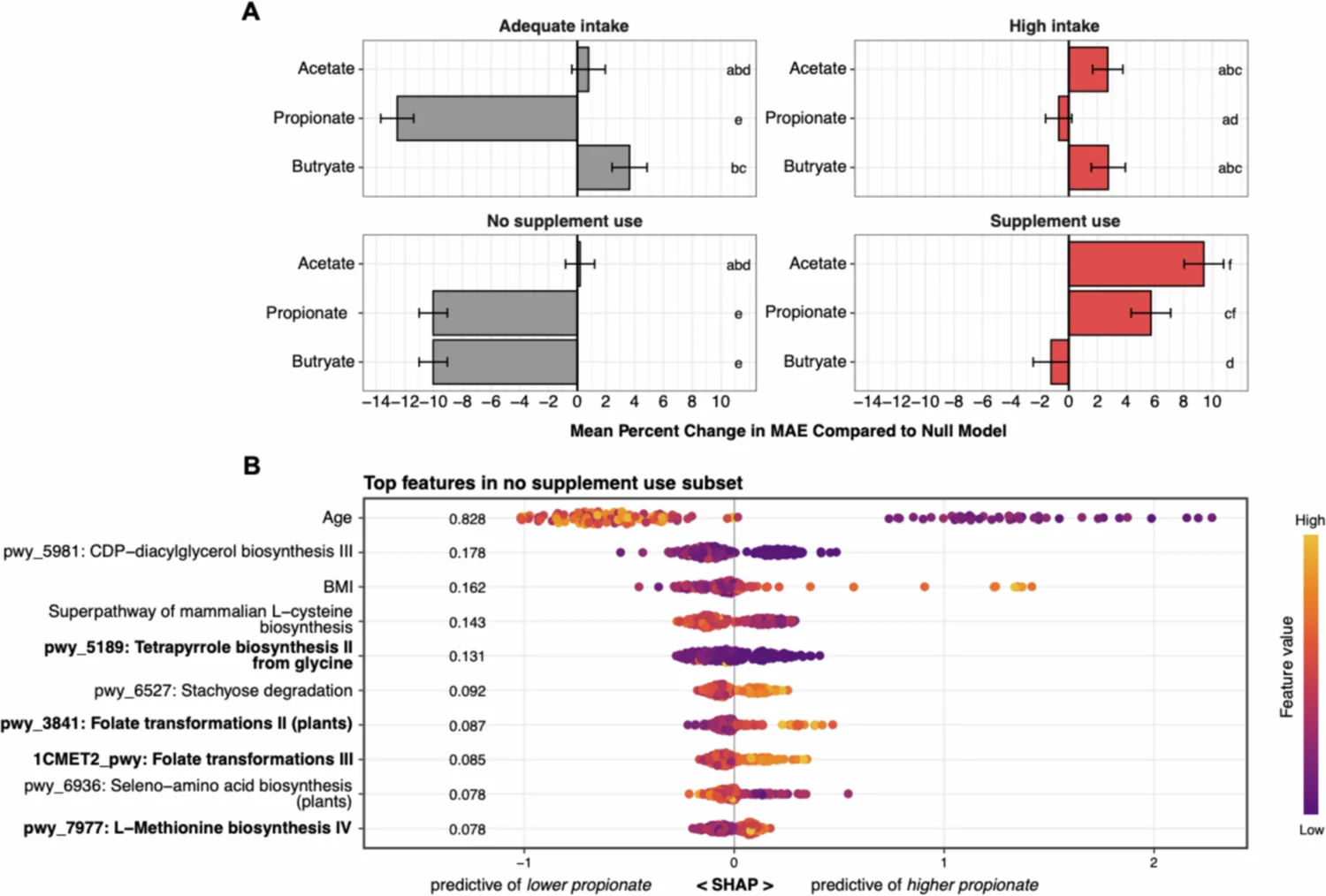
Predicting fecal SCFA concentrations in vitamin B_12_ dietary groups using machine learning. Bar charts depict the (A) mean percent change in the mean absolute error (MAE) between a trained model and a null model for predicting fecal SCFA concentrations from microbiome pathway abundance features. The solid vertical black line at x = 0 represents no difference in predictive accuracy between the trained and null models, and a lower mean percent change in MAE represents a more accurate model. The error bars represent the standard error of the mean (*n* = 100). Differences in means were determined using Dunn's test, where non-matching letters represent significant differences in means. (B) The Beeswarm plot shows the top pathway abundance features in a random forest regression model predicting fecal propionate in the subset of participants who do not use vitamin B_12_ supplements. Points are colored by their respective feature value. Pathway features in bold are related to one-carbon metabolism.

Having established that B_12_ intake is associated with fecal SCFA concentrations, we constructed random forest models within B_12_ intake subgroups (adequate intake, high intake, supplement use, and no supplement use) to examine whether microbial pathway and taxonomic abundances could predict fecal SCFA concentrations in different dietary contexts, thereby exploring potential microbial associations with B_12_ intake (Supplemental Table 5). In general, we found that propionate was frequently the most accurately predicted fecal SCFA, regardless of whether the models used pathway abundance or taxonomic composition features (Figure 6A, Supplemental Figure 4).

Among the models using pathway abundance features, the prediction of fecal propionate was restricted to the adequate intake group and no supplement use groups, while the prediction of fecal butyrate was restricted to the no supplement use group. We further investigated the pathway features that drove the top models for predicting fecal propionate. In the no supplement use group, the top features included both person-specific and microbial predictors. Among the person-specific features, younger age and higher BMI were associated with predictions of higher fecal propionate concentrations. Among the microbial features, several top predictors were related to one-carbon metabolism. Namely, greater pathway abundances of two fotate transformation pathways linked to plants (pathway 3841) and *E. coli* (1CMET2) were associated with higher predictions of fecal propionate concentrations (Figure 6B). Similarly, the biosynthesis of the amino acid methionine surfaced as a top feature predicting greater fecal propionate concentrations in the no supplement use group. Conversely, a greater abundance of the tetrapyrrole biosynthesis II from the glycine pathway was associated with lower predictions of fecal propionate concentrations in this group.

Overall, in terms of predictive accuracy, we found that taxonomic-based models supported our pathway-based results. Among the microbial relative abundance feature set, accurate prediction of propionate and butyrate was widespread throughout all subsets of participants based on B_12_ intake (Supplemental Figure 4A). Among the top predictors, higher abundances of *Oscillospiraceae* and *Eggerthellaceae* were consistently associated with lower fecal propionate concentrations across all subgroups (Supplemental Table 9). In addition, several recognized or putative butyrogenic taxa, including *Agathobacter rectalis*, *Phocaeicola vulgatus*, *Bacteroides ovatus*, *Anaerostipes hadrus* A, and *Lachnospiraceae* AM51-8, were all positively associated with fecal butyrate concentrations in a model predicting butyrate in the no supplement use group (Supplemental Table 9).

## Discussion

This study examined the associations of B_12_ intake from foods and supplements with plasma B_12_ concentrations, gut microbiome composition, pathway abundances, and fecal and plasma SCFA concentrations in 240 healthy U.S. adults from the USDA Nutritional Phenotyping Study. As expected, plasma B_12_ concentrations were positively correlated with the intake of B_12_ from food and supplements.^62^ All participants consumed adequate B_12_ and nearly all were B_12_ replete. Yet, there were notable differences in gut microbial functional capacity and fecal SCFAs levels between adequate and high consumers.

Assembly-based metagenomics revealed that approximately 20% of gut microbial genomes per subject encode a complete suite of B_12_ biosynthetic genes, consistent with prior estimates.^63^ In humans, geography is associated with differences in B_12_ biosynthetic pathway abundance, while dietary differences within a single country are not.^64^ We found that microbial B_12_ biosynthetic potential in healthy U.S. adults was reduced in those with high B_12_ intake. As this was a healthy cohort, the long-term implications of reduced microbial biosynthesis are unknown. Research in animals suggests that excessive dietary supplementation of B_12_ is associated with less resilience to enteric challenge.^65^ Additionally, the participants in this study were almost entirely B_12_ replete, and the relationship between diet and microbes would potentially differ in a markedly B_12_-deficient state,^66^ underscoring the importance of evaluating both extremes of nutrient exposure.

We found that habitual consumption of greater than 8.51  µg per day of dietary B_12_ and B_12_ supplementation was associated with lower fecal SCFA concentrations. Our models controlled for soluble fiber and dietary methionine, as these factors would likely impact the available fermentable substrates for microbial production of SCFAs and are purported modulators of fecal SCFA production that are co-consumed with B_12_ ^20^ , respectively. The significance of the relationship between B_12_ and fecal SCFAs despite adjustment for dietary pattern (soluble fiber, dietary methionine) suggests that isolated nutrients rather than broad dietary patterns may shape microbial metabolite production. This aligns with reports that dietary fiber interventions alone often fail to elicit sustained changes in fecal SCFA profiles.^67^ ^,^ ^68^ Our results differ from murine work showing that B_12_ supplementation had no effect on cecal SCFAs^65^ ^,^ ^69^ and culture models showing no effect with B_12_ ^23^ or even positive effects with cobalamin supplementation on SCFA concentrations.^70^ However, our results are consistent with evidence that both high-methionine diets and animal-rich dietary patterns are linked to reduced SCFA production.^71^ ^,^ ^72^ The inconsistent findings in animal studies could be due to the variable baseline gut microbes in those studies and the amount of fermentable substrates in their diet.

Reduced fecal SCFA concentrations are associated with states of dysbiosis in inflammatory bowel disease,^73^ ^,^ ^74^ colorectal cancer,^75^ diabetes,^76-78^ kidney disease,^79^ and hypertension.^80^ SCFAs, especially butyrate, are vitally important to many aspects of human health. Given our finding that dietary B_12_, in excess of 8.51  µg per day, is associated with lower fecal SCFAs in healthy adults, an intervention trial is sorely needed to determine if there should be an upper limit on B_12_ consumption.

Unlike fecal SCFAs, there was no association of dietary or supplementary B_12_ with plasma SCFAs. Others have noted that fecal SCFAs, but not plasma SCFAs, significantly associate with the gut microbiome.^26^ ^,^ ^81^ This may be due to the metabolism of SCFAs by intestinal cells and the liver, resulting in low levels of SCFAs that appear in peripheral blood.^82^ ^,^ ^83^ Mechanistic evidence for the effect of SCFAs on health has been mostly demonstrated by their local action in the gut, while the interplay with extraintestinal sites is less well-understood (reviewed in^84^). Therefore, even if higher B_12_ intake does not have measurable effects on peripheral SCFAs, the effect of higher B_12_ intake on gastrointestinal health and mucosal immunity via microbially-produced SCFAs is worth further pursuit.

Using machine learning models, we found fecal propionate concentrations were predictable using specific microbial metabolic pathways in participants whose B_12_ intake ranged from 2.4—8.51 µg per day or who did not use B_12_ supplements. Important features in a model specific to participants who did not use B_12_ supplements were related to one-carbon metabolism that converged on methionine synthesis. We propose that the association between methionine biosynthesis pathway abundance and fecal propionate concentrations in this group arose because methionine synthase (MetH) requires B_12_ as an enzymatic cofactor.^63^ In taxa that lack a B_12_-independent alternative, luminal B_12_ deficiency has the potential to prevent microbes from synthesizing methionine.^85^ ^,^ ^86^ However, when B_12_ is scarce, some microbes can instead synthesize methionine via a B_12_-independent pathway (MetE) that uses a derivative of folate as a methyl donor.^87^ Thus, methionine biosynthesis and folate transformation pathway abundances may reflect the consumption of methionine from the luminal environment. The bacterial biosynthesis of methionine via folate-dependent mechanisms (MetE) may potentiate further microbial propionate production in these individuals. Further, less tetrapyrrole biosynthesis (from glycine) pathway abundance was predictive of higher fecal propionate. If this reflects a reduction in B_12_ synthesis, it would be consistent with a shift toward folate-dependent methionine synthesis, as this pathway results in delta-aminolevulinic acid, the precursor to anaerobic B_12_ biosynthesis.^88^ We propose that methionine biosynthesis and related pathways reflect fluxes in one-carbon metabolism, influencing the availability of intermediates that are channeled toward propionate synthesis in the microbiome.

Conversely, the machine learning models suggest that SCFA production is insensitive to microbial function when B_12_ intake is excessive. Given that the taxonomic composition did not differ between the adequate and high intake groups, we speculate that excess dietary B_12_ possibly suppresses intracellular microbially-produced B_12_, which, in turn, could reduce propionate production. *Bacteroides*, a major gut commensal, has been shown to have sufficiently high affinity for B_12_ that it can strip B_12_ from human intrinsic factor,^89^ suggesting that dietary B_12_ can be available to microbes in the colon. Secondly, bacterial riboswitches are known to regulate gene expression in response to availability of exogenous B_12_ analogues^90^ and can suppress B_12_ uptake^91^ and synthesis.^92^ Therefore, its possible that higher exogenous B_12_ results in a scarcity of intracellular B_12_, thereby reducing propionate production.

Our study has several notable strengths. First, through strategic participant recruitment, the parent observational cohort evenly covered all age groups, both sexes, and a broad range of BMIs. This balance was largely retained in the present analysis, with the exception of underrepresentation of participants in the highest BMI category (≥30 kg/m^2^), which we acknowledge as a limitation below. Nonetheless, the demographic breadth of these data minimized bias that may have otherwise been introduced to the study, given the influence of person-specific factors on fecal SCFA concentrations.^93^ ^,^ ^94^ Second, we employed deep shotgun metagenomic sequencing, which enabled a direct assessment of microbial pathways rather than relying on inferred functions from amplicon-based sequencing. This approach provides higher taxonomic and pathway-level resolution, addressing a key limitation in the current literature. Third, we integrated metagenomic data with dietary intake data, allowing us to examine the relationships between host diet and the gut microbial community, with verification of B_12_ status by direct measurement in plasma. Finally, we adopted a machine learning framework to explore microbial associations with fecal SCFA concentrations. Leveraging these emerging computational tools can facilitate hypothesis generation, benefitting future diet and microbiome research.

However, this study comes with notable limitations. Firstly, the observational study design precludes causal inference. We attempt to address this limitation by using multiple and generalized regression modeling to mitigate potential selection bias and confounding factors that can influence dependent outcomes.^95^ Secondly, as our sample size is limited (*n* = 277), caution should be exercised in generalizing our findings to other human cohorts. This work investigated a secondary objective of the parent study, which was not explicitly powered to explain the variation in SCFAs in response to dietary B_12_. For the machine learning work, we attempt to mitigate this limitation through bootstrapping to estimate model performance variability associated with the initial test-train splits, and report the error associated with our model performance.^96^ Thirdly, in a cross-sectional study, we are limited to a single “snapshot” in time regarding our outcomes of interest. Also, while food frequency questionnaires are a cost- and time-effective way to estimate usual intake over long periods, they are subject to recall bias.^97^ Finally, the underrepresentation of participants in the highest BMI category (≥30 kg/m^2^) in our analysis introduces the possibility that our findings may not generalize to adults with obesity who are otherwise healthy.

In summary, our findings suggest that high B_12_ intake, whether from foods or supplements, has fecal SCFA associations in humans, such as those observed with high methionine in animals. Consistent with evidence from animal models that demonstrate excess dietary B_12_ can perturb the gut microbiome, our results indicate that while structural changes in microbial composition appear limited, higher B_12_ intake may exert functional consequences related to fecal SCFA production. Future trials in humans are warranted to test the effects of a diet replete with fermentable fiber while high in B_12_ (>8.51 µg/day) on microbial corrinoid metabolism and fecal SCFA production. As dietary guidance currently provides no upper limit on vitamin B_12_ intake, our study suggests that such an investigation is urgently needed.

## Supplementary Material

Supplemental table 9.csv

Supplemental tables and figures.docx Supplemental tables and figures.docx

## Data Availability

Metagenomic reads are deposited in the NCBI Sequence Read Archive under two accession numbers: PRJNA795985 and PRJNA1090850. Requests for non-metagenomic data from the USDA Agricultural Research Service Western Human Nutrition Research Center (WHNRC) Nutritional Phenotyping Study used in this analysis should be made via an email to the senior WHNRC author on the publication of interest. Requests will be reviewed quarterly by a committee consisting of the study investigators. Code to reproduce our results is available in a public GitHub repository: https://github.com/margarethilliard/VB12-analysis.
