# Supplementary material for: High dietary B12 is associated with reduced gut microbial B12 biosynthesis capacity and lower fecal short-chain fatty acids in healthy United States adults
Source: Gut Microbes. 2026 Aug 21;18(1):2718567. doi: 10.1080/19490976.2026.2718567 (PMC13502008; doi:10.1080/19490976.2026.2718567)
Supplement: Supplementary file 1 [file KGMI_A_2718567_SM7720.docx]

**Supplemental Tables and Figures**

| **Characteristic** | **N = 277**^1^ | **p-value** |
| --- | --- | --- |
| **Age group (years)** |  | 0.989 |
| 18-33 | 91 (33%) |  |
| 34-49 | 93 (34%) |  |
| 50-65 | 92 (33%) |  |
| **BMI category (kg/m²)** |  | 0.017 |
| <25 | 103 (37%) |  |
| 25-29.9 | 104 (38%) |  |
| ≥30 | 70 (25%) |  |
| **Sex** |  | 0.857 |
| Female | 137 (49%) |  |
| Male | 140 (51%) |  |
| ^1^n (%) | | |

**Supplemental Table 1:** Balance of participant characteristics from this subset of the USDA Nutritional Phenotyping study (*n* = 277). Age and BMI are grouped into clinically relevant categories consistent with the parent study. Data are presented as *n* (%). Goodness-of-fit chi-square tests were used to assess whether participants were equally distributed across categories within each variable.

| **Characteristic** | **Overall**  N = 277^1^ | **High**  N = 140^1^ | **Adequate**  N = 137^1^ | **p-value**^2^ |
| --- | --- | --- | --- | --- |
| **Age (years)** | 41 (14) | 41 (14) | 41 (13) | 0.8 |
| **Sex** |  |  |  | 0.11 |
| Female | 137 (49%) | 62 (44%) | 75 (55%) |  |
| Male | 140 (51%) | 78 (56%) | 62 (45%) |  |
| **BMI (kg/m²)** | 27.0 (4.7) | 27.3 (4.9) | 26.8 (4.5) | 0.5 |
| ^1^Mean (SD); n (%) | | | | |
| ^2^Welch Two Sample t-test; Pearson's Chi-squared test | | | | |

**Supplemental Table 2:** Baseline participant characteristics based on adequate (2.4 - 8.51 µg/day) or high (> 8.51 µg/day) vitamin B_12_ intake. Continuous variables are presented as mean (SD) and compared using independent t-tests. Categorical variables are presented as *n* (%) and compared using chi-square tests. P-values represent between-group differences.

| **Characteristic** | **Overall**  N = 277^1^ | **No**  N = 156^1^ | **Yes**  N = 121^1^ | **p-value**^2^ |
| --- | --- | --- | --- | --- |
| **Age (years)** | 41 (14) | 41 (14) | 42 (14) | 0.6 |
| **Sex** |  |  |  | 0.3 |
| Female | 137 (49%) | 72 (46%) | 65 (54%) |  |
| Male | 140 (51%) | 84 (54%) | 56 (46%) |  |
| **BMI (kg/m²)** | 27.0 (4.7) | 26.8 (4.3) | 27.4 (5.2) | 0.3 |
| ^1^Mean (SD); n (%) | | | | |
| ^2^Welch Two Sample t-test; Pearson's Chi-squared test | | | | |

**Supplemental Table 3:** Baseline participant characteristics based on vitamin B_12_ supplement use. Continuous variables are presented as mean (SD) and compared using independent t-tests. Categorical variables are presented as n (%) and compared using chi-square tests. P-values represent between-group differences.

| **K_number** | **Description** |
| --- | --- |
| K13542 | uroporphyrinogen III methyltransferase / synthase [EC:2.1.1.107 4.2.1.75] |
| K13540 | precorrin-2 C20-methyltransferase / precorrin-3B C17-methyltransferase [EC:2.1.1.130 2.1.1.131] |
| K02302 | uroporphyrin-III C-methyltransferase / precorrin-2 dehydrogenase / sirohydrochlorin ferrochelatase [EC:2.1.1.107 1.3.1.76 4.99.1.4] |
| K02189 | cobalt-precorrin 5A hydrolase [EC:3.7.1.12] |
| K03394 | precorrin-2/cobalt-factor-2 C20-methyltransferase [EC:2.1.1.130 2.1.1.151] |
| K03399 | cobalt-precorrin-7 (C5)-methyltransferase [EC:2.1.1.289] |
| K02227 | adenosylcobinamide-phosphate synthase [EC:6.3.1.10] |
| K22011 | sirohydrochlorin cobalto/nickelchelatase [EC:4.99.1.3 4.99.1.11] |
| K05895 | precorrin-6A/cobalt-precorrin-6A reductase [EC:1.3.1.54 1.3.1.106] |
| K06042 | precorrin-8X/cobalt-precorrin-8 methylmutase [EC:5.4.99.61 5.4.99.60] |
| K02304 | precorrin-2 dehydrogenase / sirohydrochlorin ferrochelatase [EC:1.3.1.76 4.99.1.4] |
| K02226 | alpha-ribazole phosphatase [EC:3.1.3.73] |
| K24866 | precorrin-2 dehydrogenase [EC:1.3.1.76] |
| K02231 | adenosylcobinamide kinase / adenosylcobinamide-phosphate guanylyltransferase [EC:2.7.1.156 2.7.7.62] |
| K21479 | cobalt-factor III methyltransferase [EC:2.1.1.272] |
| K00595 | precorrin-6B C5,15-methyltransferase / cobalt-precorrin-6B C5,C15-methyltransferase [EC:2.1.1.132 2.1.1.289 2.1.1.196] |
| K13541 | cobalt-precorrin 5A hydrolase / cobalt-factor III methyltransferase / precorrin-3B C17-methyltransferase [EC:3.7.1.12 2.1.1.272 2.1.1.131] |
| K03795 | sirohydrochlorin cobaltochelatase [EC:4.99.1.3] |
| K02496 | uroporphyrin-III C-methyltransferase [EC:2.1.1.107] |
| K00589 | uroporphyrin-III C-methyltransferase [EC:2.1.1.107] |
| K19221 | cob(I)alamin adenosyltransferase [EC:2.5.1.17] |
| K02233 | adenosylcobinamide-GDP ribazoletransferase [EC:2.7.8.26] |
| K05934 | precorrin-3B C17-methyltransferase / cobalt-factor III methyltransferase [EC:2.1.1.131 2.1.1.272] |
| K02190 | sirohydrochlorin cobaltochelatase [EC:4.99.1.3] |
| K02303 | uroporphyrin-III C-methyltransferase [EC:2.1.1.107] |
| K02191 | cobalt-precorrin-6B (C15)-methyltransferase [EC:2.1.1.196] |
| K02224 | cobyrinic acid a,c-diamide synthase [EC:6.3.5.9 6.3.5.11] |
| K00768 | nicotinate-nucleotide--dimethylbenzimidazole phosphoribosyltransferase [EC:2.4.2.21] |
| K22316 | ribonuclease H / adenosylcobalamin/alpha-ribazole phosphatase [EC:3.1.26.4 3.1.3.73] |
| K02188 | cobalt-precorrin-5B (C1)-methyltransferase [EC:2.1.1.195] |
| K13543 | uroporphyrinogen III methyltransferase / synthase [EC:2.1.1.107 4.2.1.75] |
| K00798 | cob(I)alamin adenosyltransferase [EC:2.5.1.17] |
| K02232 | adenosylcobyric acid synthase [EC:6.3.5.10] |
| K05936 | precorrin-4/cobalt-precorrin-4 C11-methyltransferase [EC:2.1.1.133 2.1.1.271] |
| K02225 | cobalamin biosynthesis protein CobC |

**Supplemental Table 4:** Enzymes used to create a custom metabolic module for use with anvi'o8. These enzymes were used to profile metagenome assembled genomes for their genomic capability for anaerobic vitamin B_12_ biosynthesis.

| **predictor dataset** | **response** | **subset** | **software** |
| --- | --- | --- | --- |
| composition | acetate | high B_12_ intake | TaxaHFE-ML |
| composition | propionate | high B_12_ intake | TaxaHFE-ML |
| composition | butyrate | high B_12_ intake | TaxaHFE-ML |
| pathways | acetate | high B_12_ intake | DietML |
| pathways | propionate | high B_12_ intake | DietML |
| pathways | butyrate | high B_12_ intake | DietML |
| composition | acetate | adequate B_12_ intake | TaxaHFE-ML |
| composition | propionate | adequate B_12_ intake | TaxaHFE-ML |
| composition | butyrate | adequate B_12_ intake | TaxaHFE-ML |
| pathways | acetate | adequate B_12_ intake | DietML |
| pathways | propionate | adequate B_12_ intake | DietML |
| pathways | butyrate | adequate B_12_ intake | DietML |
| composition | acetate | supplement users | TaxaHFE-ML |
| composition | propionate | supplement users | TaxaHFE-ML |
| composition | butyrate | supplement users | TaxaHFE-ML |
| pathways | acetate | supplement users | DietML |
| pathways | propionate | supplement users | DietML |
| pathways | butyrate | supplement users | DietML |
| composition | acetate | supplement non-users | TaxaHFE-ML |
| composition | propionate | supplement non-users | TaxaHFE-ML |
| composition | butyrate | supplement non-users | TaxaHFE-ML |
| pathways | acetate | supplement non-users | DietML |
| pathways | propionate | supplement non-users | DietML |
| pathways | butyrate | supplement non-users | DietML |

**Supplemental Table 5:** Machine learning models (*n* = 24) predicting fecal SCFAs from either gut microbial compositional abundance (composition) or pathway abundance (pathway) data. All models controlled for the covariates age, sex, BMI, and soluble fiber intake.

| **predictor** | **response** | **p-value** |
| --- | --- | --- |
| intake group | fecal acetate | 0.0002554 |
| intake group | fecal propionate | 0.02451 |
| intake group | fecal butyrate | 0.03788 |
| supplement use | fecal acetate | 0.01066 |
| supplement use | fecal propionate | 0.03866 |
| supplement use | fecal butyrate | 0.03641 |

**Supplemental Table 6:** Associations between individual fecal SCFA concentrations and dietary vitamin B_12_ intake groups. P-values represent between-group differences that were calculated using the Mann-Whitney U test, unadjusted for covariates.

| **response** | **predictor** | **model type** | **covariates** | **p-value** |
| --- | --- | --- | --- | --- |
| plasma acetate | intake group (categorical; adequate/high) | Mann-Whitney U test | N/A | 0.7063 |
| plasma propionate | intake group (categorical; adequate/high) | Mann-Whitney U test | N/A | 0.7446 |
| plasma butyrate | intake group (categorical; adequate/high) | Mann-Whitney U test | N/A | 0.5134 |
| plasma acetate | supplement use (categorical; yes/no) | Mann-Whitney U test | N/A | 0.713 |
| plasma propionate | supplement use (categorical; yes/no) | Mann-Whitney U test | N/A | 0.9972 |
| plasma butyrate | supplement use (categorical; yes/no) | Mann-Whitney U test | N/A | 0.7656 |
| plasma acetate | habitual B_12_ intake (continuous) | Partial Spearman correlation | age, sex, BMI, dietary fiber, dietary methionine | 0.7581495 |
| plasma propionate | habitual B_12_ intake (continuous) | Partial Spearman correlation | age, sex, BMI, dietary fiber, dietary methionine | 0.883585 |
| plasma butyrate | habitual B_12_ intake (continuous) | Partial Spearman correlation | age, sex, BMI, dietary fiber, dietary methionine | 0.2521076 |
| plasma acetate | intake group (categorical; adequate/high) | Linear regression (normalized response) | age, sex, BMI, dietary fiber, dietary methionine | 0.4355888 |
| plasma acetate | supplement use (categorical; yes/no) | Linear regression (normalized response) | age, sex, BMI, dietary fiber, dietary methionine | 0.4559747 |
| plasma propionate | intake group (categorical; adequate/high) | Tobit regression | age, sex, BMI, dietary fiber, dietary methionine | 0.7130619 |
| plasma propionate | supplement use (categorical; yes/no) | Tobit regression | age, sex, BMI, dietary fiber, dietary methionine | 0.7158622 |
| plasma butyrate | intake group (categorical; adequate/high) | Tobit regression | age, sex, BMI, dietary fiber, dietary methionine | 0.2209964 |
| plasma butyrate | supplement use (categorical; yes/no) | Tobit regression | age, sex, BMI, dietary fiber, dietary methionine | 0.6206908 |

**Supplemental Table 7:** Associations between dietary B_12_ and plasma SCFA concentrations. Plasma acetate was Box-Cox transformed in linear regression models. Tobit regression lower censoring thresholds were set to the lowest response variable value. P-values for covariate-adjusted models represent the associations between the predictor and response variable after covariate adjustment, if applicable. Since no statistically significant associations were detected across these analytical approaches, p-values are not corrected for false discovery.

| **Diversity Metric** | **Predictor** | **R-squared** | **P value** |
| --- | --- | --- | --- |
| shannon | supplement use | 0.001 | 0.723272727 |
| invsimpson | supplement use | 0.001 | 0.723272727 |
| richness | supplement use | 0.005 | 0.452 |
| shannon evenness | supplement use | 0.003 | 0.521333333 |
| simpson evenness | supplement use | 0.003 | 0.521333333 |
| pielou evenness | supplement use | 0.003 | 0.521333333 |
| shannon | intake group | 0 | 0.895 |
| invsimpson | intake group | 0.006 | 0.452 |
| richness | intake group | 0.007 | 0.452 |
| shannon evenness | intake group | 0.017 | 0.1788 |
| simpson evenness | intake group | 0.009 | 0.452 |
| pielou evenness | intake group | 0.017 | 0.1788 |
| invsimpson | normalized B12 intake (continuous) | 0.0103 | 0.13695 |
| pielou evenness | normalized B12 intake (continuous) | 0.014 | 0.13695 |
| richness | normalized B12 intake (continuous) | 0.00406 | 0.3492 |
| shannon | normalized B12 intake (continuous) | 0.000114 | 0.86 |
| shannon evenness | normalized B12 intake (continuous) | 0.014 | 0.13695 |
| simpson evenness | normalized B12 intake (continuous) | 0.0104 | 0.13695 |
| bray | supplement use | 0.003 | 0.827 |
| bray | intake group | 0.004 | 0.4466667 |
| bray | normalized B12 intake (continuous) | 0.004 | 0.4466667 |
| jaccard | supplement use | 0.003 | 0.827 |
| jaccard | intake group | 0.004 | 0.4466667 |
| jaccard | normalized B12 intake (continuous) | 0.004 | 0.4466667 |

**Supplemental Table 8:** Gut microbiome alpha and beta diversity metrics compared between dietary groups based on B_12_ intake. P-values were adjusted for multiple comparisons using the False Discovery Rate method.

**Supplemental Figure 1:** STROBE diagram for this analysis of the USDA Nutritional Phenotyping study. Final analytic sample sizes used are in bold.

**Supplemental Figure 2**: Partial regressions between plasma B_12_ concentration and **(A)** age, **(B)** BMI, **(C)** or sex while adjusting for co-variates in the model. Points represent individual participants, dashed lines are the fitted linear relationship, and the shaded area represents the 95% confidence interval. Upper and lower lines of the boxplots represent the upper and lower quartiles, while the middle line represents the median. Differences in means were determined using a Wilcoxon rank sum test and significant is denoted by asterisks (∗ *p* < 0.05, ∗∗ *p* < 0.01, ∗∗∗ *p* < 0.001; ns = not significant). **(D)** The table contains the estimated coefficients, 95% confidence intervals, and p-values from a linear regression model predicting plasma B_12_ concentrations from habitual dietary B_12_ intake, BMI, age, and sex.

**Supplemental Figure 3**: An overview of the relationship between B_12_ and methionine intake in a healthy U.S. adult cohort. Partial regressions show the relationships between dietary B_12_ and methionine in either the **(A)** adequate and high B_12_ intake groups or **(C)** the B_12_ supplement use and no B_12_ supplement use groups. All partial regressions adjust for age, sex, BMI, and soluble fiber intake in the models. Solid colored lines represent the linear line of best it, and the shaded area represents the 95% confidence interval. Colors of points and lines are based on B_12_ intake group, or B_12_ supplement use. Boxplots comparing dietary methionine intake based on **(B)** participant intake of vitamin B_12_ relative to the 50^th^ percentile (adequate intake: 2.4 - 8.51 µg/day, high intake: > 8.51 µg/day) or **(D)** participant use of vitamin B_12_ supplements**.** Upper and lower lines of the boxplots represent the upper and lower quartiles, while the middle line represents the median. Black diamonds represent the mean intake in each group. Differences between group means were determined by using a Wilcoxon rank sum test and significance is denoted by asterisks (∗ *p* < 0.05, ∗∗ *p* < 0.01, ∗∗∗ *p* < 0.001; ns = not significant).

**Supplemental Figure 4**: Model performance metrics predicting fecal SCFA concentrations using machine learning. The bar charts depict **(A)** the mean percent change in MAE between a trained model and a null model, or the **(B)** average explained variance (R^2^) for models that predict fecal SCFA concentrations from relative abundances of microbial taxa **(C)** or pathway abundances from subsets of data based on dietary B_12_ intake group (adequate intake: 2.4 - 8.51 µg/day, high intake: > 8.51 µg/day) and B_12_ supplement use. The solid vertical black line a x=0 represents no difference in predictive accuracy between a trained and null model, and a lower mean percent change in MAE represents a more accurate model. Error bars represent the standard error of the mean. Differences in means were determined using Dunn's test, where non-matching letters represent significant differences in means.
